# P350: Study on the application of standards of quality emergency obstetric neonatal in Ghana

**DOI:** 10.1186/2047-2994-2-S1-P350

**Published:** 2013-06-20

**Authors:** Ellis O Dabo, N Affodo, I Adams

**Affiliations:** 1 Kumasi Centre for Collaborative Research in Tropical Medicine, Accra, Ghana; 2 Ministry of Health, Accra, Ghana

## Introduction

Quality health system is not only an ideological way of delivering health service in many a country but involves the practical systems and institutions to deliver. Over the last two decades issues to do with quality of health care has been phenomenally on the increase with the population demanding quality health care from those who deliver them.

## Objectives

The purpose of this study therefore was to do a baseline analysis of the existence of quality health care initiatives in selected hospitals in Kumasi, Ashanti Region as a field site for Ghana.

## Methods

Ghana like many other countries in the West African subregion has implemented quality health care over this period of time. The study was supported by RIPAQS and the West Africa Health Organization (WAHO), with additional support from the Governments of member states of the ECOWAS.

The study was carried out over a period of June to July, 2012, involving face to face administration of questionnaires that were pretested in selected hospitals in Ghana after an initial pilot in Abidjan the Ivorian capital. 359 different categories of health staff from six purposively selected health facilities were sampled for the study.

## Results

Findings reveal that quality health systems have been implemented largely in many of the health facilities in Ghana over the years. Many of the indicators of quality health care assessed covering areas such as operational, technical and institutional dimensions have been fully implemented. Only a few of the indicators had either been partially implemented or non-existent. A few were either under discussion or had no idea of actions in place. There appeared to be a general loss of vigour and zest for sustained quality healthcare to be implemented within the selected health facilities. Factors that are thought to be impeding the process has to do mainly with lack of funding, lack of communication and in some instances institutional leadership have prevented in many instances the implementation and role out of such plans.

## Conclusion

The study recommends the implementation of a sustained strategy for implementing quality health care in particular all the initiatives that are in the draft stage. The renewed call for managers of quality health systems within local health budget management centres must be encouraged.

## Disclosure of interest

None declared

